# *Nocardia mikamii* a Novel Species Causing Disseminated Nocardiosis: A Literature Review of Disseminated Nocardiosis

**DOI:** 10.1155/2014/869153

**Published:** 2014-10-16

**Authors:** Muhammad Khan, Mohammed Muqeet Adnan, Najmi Shahbaz, Muhammad Hamza, Sufyan Abdul Mujeeb

**Affiliations:** ^1^Department of Medicine, University of Oklahoma Health Sciences Center, Oklahoma City, OK 73117, USA; ^2^Dow Medical College, Dow University of Health Sciences, Karachi 74200, Pakistan; ^3^University of Illinois at Chicago, Chicago, IL 60612, USA

## Abstract

*Nocardia* is an uncommon Gram-positive organism. It typically appears as delicate filamentous Gram-positive branching rods. In the United States it was estimated to be approximately 500 to 1000 new cases per year. The organism causes disease in immunocompromised individuals with pulmonary infection representing the most common site of infection. *Nocardia mikamii* has been a recently isolated pathogen and not many cases of disseminated infection with this organism has been reported in the literature; we present a case of disseminated nocardiosis (mikamii sp.) in an immunocompromised patient. We also present a literature review on nocardiosis.

## 1. Case Description

**Figure 1:**
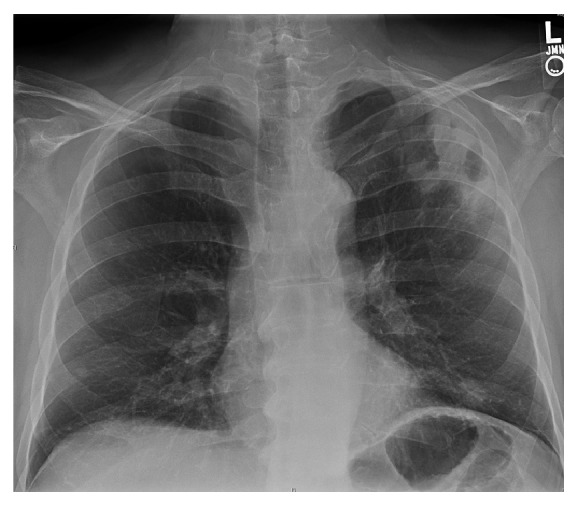
Chest X-ray at day of admission.

A 59-year-old Caucasian male presented to emergency department with left sided chest pain and weakness for three weeks. Patient was in usual state of health approximately 3 weeks back when he started having intermittent left sided chest pain, nonexertion, nonradiating, 3/10 intensity, sharp in quality, and only localized to left upper chest, aggravated by deep breathing and movement. Patient also reported progressive fatigue and shortness of breath around the same time. He had past medical history of idiopathic thrombocytopenic purpura (ITP) diagnosed 6 weeks back and was on prednisone at the time of presentation. Review of system was remarkable for progressive nonproductive cough and diffuse headache. On admission his Temp = 36.7°C, *P* = 77/min, BP: 140/79 mmHg, RR = 14/min, and saturation 98% on 2L nasal cannula. Examination was remarkable for increased tactile vocal fremitus on left lung with bronchial breath sounds and egophony. Labs showed WBC = 15.3 K/cc^3^, Hb = 14.7 mmHg, and Plt = 120 K/cc^3^, while his electrolytes were normal. Chest X-ray on admission showed left upper consolidation (see Figure 1).

**Figure 2:**
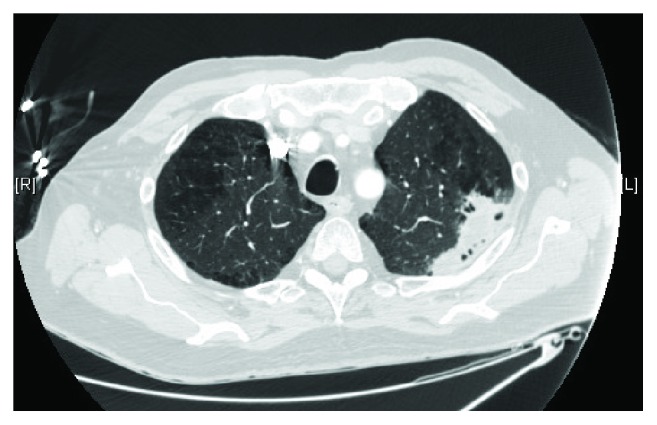
CT chest on presentation.

CT chest showed consolidative opacity in the posterolateral left upper lobe with areas of cavitation suggesting necrosis along with small cavitary lesion in the right upper lobe (see Figure 2).

**Figure 3:**
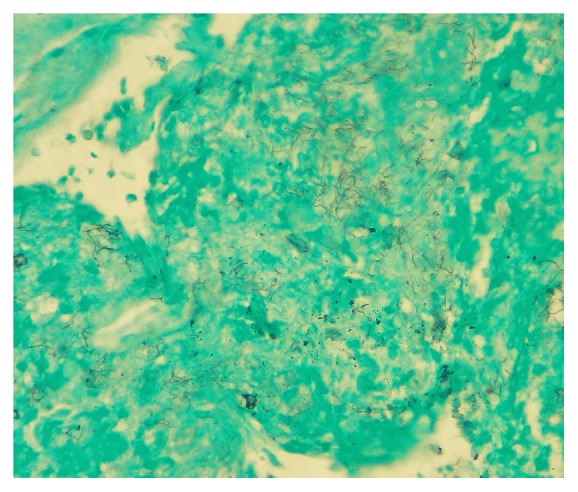
Biopsy showing filamentous bacteria.

An IR guided lung biopsy done showed numerous branching filamentous bacteria suspicious for* Nocardia mikamii* (see Figure 3).

**Figure 4:**
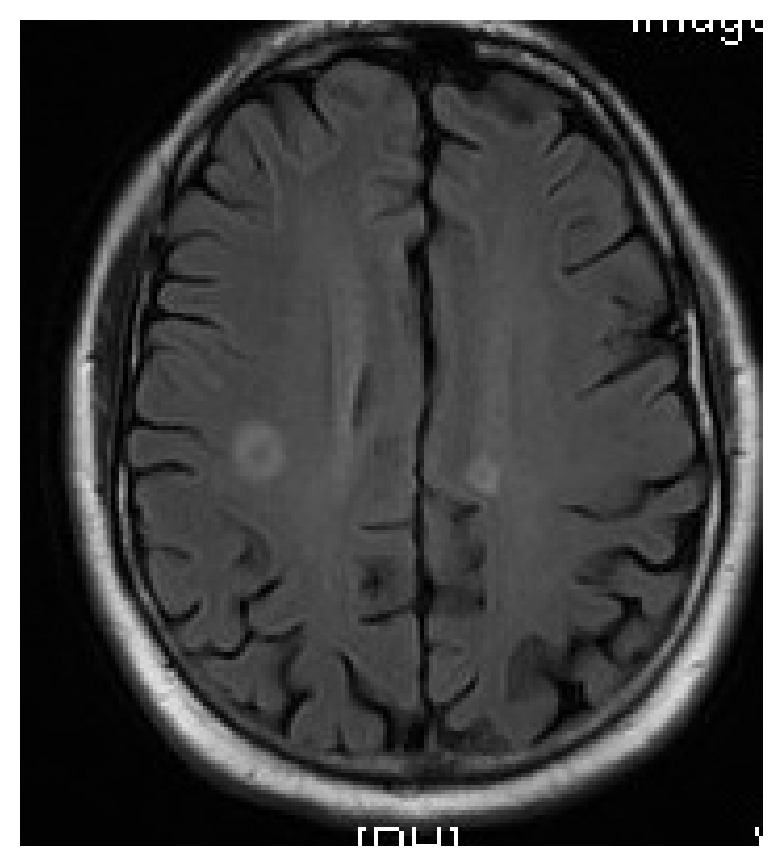
MRI brain on admission.

As the patient had headaches, MRI brain was done which showed findings consistent with multifocal small pyogenic cerebral abscesses (see Figure 4).

Patient was initially started on intravenous trimethoprim-sulfamethoxazole, intravenous imipenem, and oral linezolid. However, after 10 days linezolid was discontinued owing to a drop in platelet count. Patient was started on intravenous amikacin. Patient finished 5 weeks of induction with triple antibiotic therapy with a good clinical response. At this point bacterial susceptibilities were available which showed the bacteria were resistant to tobramycin and amoxicillin-clavulanate, but susceptible to trimethoprim-sulfamethoxazole and imipenem.

Patient was switched to oral trimethoprim-sulfamethoxazole and intravenous imipenem was continued for 5 months via home antibiotics. Patient was followed up in clinic. Repeat MRI brain showed almost resolution of disease.

## 2. Discussion

Nocardiosis is an uncommon Gram-positive bacterial infection caused by genus* Nocardia* of aerobic actinomycetes. Typically, the bacteria causes' disease in immunocompromised patients, but approximately up to one-third infection can occur in immunocompetent patients [1].

In 2010, Jannat-Khah et al. reported four cases of a unique species of genus* Nocardia* isolated from clinical respiratory sources and characterized using a polyphasic taxonomic approach. On the basis of 16S rRNA gene sequence analyses, these strains were found to be 100% similar to each other and DNA-DNA hybridization and physiological and biochemical tests supported the genotypic and phenotypic differentiation of the novel strains from related species (see Table 1). The new strain represented a novel species within the genus* Nocardia*, for which the name* Nocardia mikamii* sp. was proposed [2].

Our case is unique because of disseminated involvement of brain and lung by* Nocardia mikamii*. The patient was initially treated with five weeks induction of triple intravenous antibiotics with TMP-SMX, amikacin, and imipenem with adequate clinical response. The patient was then continued on long term oral TMP-SMX and intravenous imipenem for 5 months with near resolution of brain and lung lesion along with good clinical response.

*Nocardia* has the ability to cause localized or systemic suppurative disease in humans and animals [3, 4].* Nocardia* typically appears as delicate filamentous Gram-positive branching rods, which could be differentiated from* Actinomyces* by acid-fast staining, as* Nocardia* typically exhibits varying degree of acid fastness owing to the presence of mycolic acid in its cell wall [5]. Once considered to be fungi, cell wall molecular analysis confirmed that these organisms are actually bacteria [6].

The genus* Nocardia* includes more than 80 species, at least 33 of which are pathogenic to humans [7]. The older classification was based on biochemical properties, but classification has become more complex with the use of antibiotic susceptibility profiles and molecular techniques, such as analysis of the 16S rRNA gene, restriction fragment length polymorphism, and multilocus sequence analysis [8–11].

The organism is found worldwide in soil and aquatic environment and can become airborne particularly on dust particles. Due to this mechanism, inhalation of the bacteria is thought to be the most common route of infections [12], but infection via ingestion of contaminated food products [5], direct inoculation causing cutaneous disease [13], and in rare cases nosocomial spread of the disease has been indicated in the literature [14].

## 3. Risk Factors

As mentioned earlier, the disease is most common in immunocompromised individuals, most often with defective cell-mediated immunity [1, 4]. There is a strong association between chronic glucocorticoid therapy and nocardiosis [15–17]. Patients with malignancy, including both solid tumors and hematological malignancies, are at increased risk [5]. In one case series, 64% patients with nocardiosis had an underlying hematological malignancy, with almost half of those patients had undergone prior hematopoietic stem cell transplant [15]. Organ transplant recipient are at risk of* Nocardia* infection, with the risk being highest in first year following transplant [4]. Interesting studies in transplant recipients treated with immunosuppressive regimens lacking steroids found a significantly decreased rate of nocardial infection [4]. In a matched case-control study of 5126 organ transplant recipients, only 0.6 percent patients were found to develop* Nocardia*, with the rates highest in lung and heart transplant recipients and lowest in liver and kidney transplant recipients [17]. Nocardiosis is uncommon in HIV-infected patients and the disease only occurs in severely immunocompromised patients with median CD4 count of 35 cell/*μ*L [18]. Other conditions associated with nocardial infection include diabetes mellitus, alcoholism, chronic obstructive pulmonary disease, tuberculosis, chronic granulomatous disease, alveolar proteinosis, and tumor necrosis factor-alpha inhibitor therapy [2–5, 17, 19].

## 4. Clinical Features

*Lungs* are the primary site of infection in more than two-third of cases [4]. As* Nocardia* is not a common human flora, isolation of* Nocardia* on sputum culture is always indicative of infection. Even though most pulmonary infections are primary, the disease could disseminate to lungs from other sites such as skin and brain [20]. Pulmonary disease could manifest as acute, subacute, or chronic infection, without any disease specific symptoms [1, 3]. Symptoms of fever, night sweats, anorexia, weight loss, cough, dyspnea, hemoptysis, and pleuritic chest pain have been described commonly [1, 3]. In severe cases, patient can present with complications such as pericarditis, empyema, mediastinitis, and superior vena cava syndrome from spread of disease from lungs to adjacent organs [21–23].

*Isolated central nervous system* (CNS) involvement appears to occur in 9% of cases; however, in disseminated disease the bacteria can cause CNS infection in up to 44% of cases [5]. The signs and symptoms of CNS involvement are highly variable and nonspecific ranging from fever, headaches, and meningismus to seizures and focal neurological deficit [6, 24]. The most important finding in CNS nocardiosis is formation of a parenchymal abscess that can occur in any region of the brain [25, 26]. Clinically silent CNS nocardial lesions have been described in immunocompetent individuals [5]. CNS nocardiosis can present with symptoms of mass lesions without any associated infectious symptoms, leading to erroneous diagnosis of primary or metastatic brain malignancy prior to biopsy [27, 28].* Nocardia* meningitis is a rare manifestation of CNS nocardiosis and can occur with and without an associated brain abscess [29]. Cerebrospinal fluid (CSF) analysis can demonstrate elevated proteins and neutrophils and low CSF glucose reflecting bacterial meningitis.

*Primary cutaneous nocardiosis* can follow any puncture wound or traumatic introduction of the organism [5]. Cutaneous nocardiosis can manifest as cellulitis, pustules, pyoderma, paronychia, or localized abscess mimicking infections due to other pyogenic bacteria [3, 5]. The infection may spread to local lymph nodes and produce a lymphocutaneous or lymphonodular picture which is similar to sporotrichosis [30, 31].

*Mycetoma (Madura foot or maduromycosis)* is a chronic, deep, progressively destructive infection of skin, underlying subcutaneous tissues, fascia, bone, and muscles following trauma to hand, foot, or leg [5, 20]. The disease could be caused by a fungal infection (eumycetoma) or aerobic* Actinomycetes* (actinomycetoma) and produces an area of localized swelling containing suppurative granulomas and multiple sinus tracts extruding macroscopic colored granules [32]. Mycetoma is the only clinical form of nocardiosis regularly associated with the presence of such granules [32].* Nocardia brasiliensis* species have been associated with more fulminant skin infection, with local invasion, and in severe cases systemic dissemination [3, 5].

*Systemic or disseminated nocardiosis* is defined as identification of infection in two noncontagious sites that may or may not include pulmonary focus [3].* Nocardia* can disseminate to virtually any organ from primary pulmonary or cutaneous focus via blood stream, even though blood cultures are negative in most cases [33].

*Nocardia bacteremia* is very rare and is commonly associated with intraluminal catheter such as central venous catheters [34, 35]. In a literature review of 36 cases of* Nocardia bacteremia*, approximately 30 percent had concomitant bacteremia with other pathogens, most commonly Gram-negative organisms [34].

Posttraumatic keratitis and endophthalmitis have been described in the literature causing primary eye infections [36–39]. Other less common sites of infections include heart valves, bone, joints, and kidneys [5, 6, 40, 41].

## 5. Diagnosis

A definite diagnosis of nocardiosis includes the isolation and identification of the organism from a clinical specimen. The mean time from the development of symptoms to diagnosis ranges from 42 days to 12 months [42].

Establishing a diagnosis of nocardiosis is difficult since an invasive procedure is often required to obtain an adequate specimen and the recovery of* Nocardia* in the laboratory is difficult because of its slow growth. According to a study, 44 percent of pulmonary infections required an invasive procedure to plot down the diagnosis of nocardiosis. When an invasive procedure is performed, cultures are positive in 85–90 percent of specimens [16].

*Gram-stain studies* show* Nocardia* spp. as delicate, filamentous, sometimes beaded [2], branching Gram-modified acid-fast staining [14]. The acid-fast nature of* Nocardia* is accurately demonstrated by a modified Kinyoun procedure which substitutes 1 percent sulfuric acid for acid alcohol as a decolorizer, which allows acid-fast* Nocardia* to retain fuchsin [19].

Most routine aerobic bacterial, fungal, and mycobacterial culture media support* Nocardia*. On culture media it shows variable colonial morphology which ranges from chalky white to pigment forming orange, yellow, or brown colonies [2], usually requiring 5–21 days for growth [4, 5].

Blood cultures should be considered for patients with endovascular devices that remain febrile despite appropriate therapy.

In vitro susceptibility studies have shown that different* Nocardia* species and strains have different patterns, hence highlighting the importance of obtaining speciation and susceptibility testing of all clinical isolates [2, 3, 9, 43].

*Polymerase chain reaction* is the fastest and most accurate method for identification of* Nocardia* spp., despite being unavailable in most clinical laboratories [44]. The sensitivity and specificity of a 16S rRNA-based PCR assay was examined using 18 samples (e.g., skin biopsies, abscess material, sputum, and bronchoalveolar lavage fluid) from patients with nocardiosis diagnosed by conventional cultures and 20 clinical samples from patients with confirmed tuberculosis as negative controls [44]. All specimens from patients with nocardiosis were positive, while none of the 20 control samples were positive, confirming the sensitivity and specificity of the primers and PCR protocol [44].

*Nocardia* spp., especially* N. asteroides* complex, have a propensity to cause brain infection. In addition, all immunocompromised patients with cutaneous and/or pulmonary nocardiosis, even those without symptoms of CNS involvement, should undergo brain imaging. Immunocompetent patients with pulmonary nocardiosis should also undergo radiographic evaluation to exclude CNS infection.

## 6. Treatment

Treatment of infection depends on type of infection and antibiotic susceptibility. The data on antibiotic susceptibility is variable. In a retrospective study of 765 isolates at Center of Disease Control and Prevention (CDC) between 1995 and 2004, 42 percent strains were found resistant to trimethoprim-sulfamethoxazole (TMP-SMX) and 61 percent were resistant to sulfamethoxazole [45]. In a more recent study of 552 clinical isolates from six major medical referral center in the United States from years 2005 to 2011 only 2 percent of isolates were found to be resistant to sulfamethoxazole [2]. The most likely explanation for this discrepancy between the results of two studies could be based on differences in preparation of samples or the interpretation of the results of in vitro susceptibility testing [46].

To date no randomized clinical study is available to determine the most effective treatment. Most authorities recommend TMP-SMX as part of first line therapy for nocardiosis [3]. This consensus is based partly upon the results of few retrospective reviews indicating increased survival in nocardiosis patients treated with sulfonamide based regimen [47]. TMP-SMX has also demonstrated synergy against nocardial infection in several in vitro studies, with the optimal ratio of TMP to SMX ranging from 1 to ≤5 to a ratio of 1 to 10 [47, 48]. TMP-SMX also has additional benefit of excellent penetration into most tissue compartments, including CNS and high bioavailability [49]. The drug is available in both oral and intravenous form [47]. Drug levels should be monitored in patients with life-threatening disease and in case of treatment failure. A serum level between 100 and 150 mcg/mL is considered therapeutic concentration [3].

TMP-SMX can be used as monotherapy or as combination depending on the site and extent of the disease [3].

In case of isolated cutaneous infection, empiric monotherapy with TMP-SMX (2.5 to 5 mg/kg of the TMP component) twice daily could be used [3]. Alternative oral agents like minocycline (100 mg orally twice daily), amoxicillin-clavulanate, and doxycycline could be used as well [3].

Combination antibiotics are required in treatment of mycetoma. Surgical excision may be undertaken before the diagnosis but is usually not warranted and antibiotics alone are able to treat the condition [47]. High dose TMP-SMX (5 mg/kg of TMP component twice daily) with or without dapsone (100 mg orally daily) is commonly used for limited disease with low risk of dissemination [50]. In case of severe disease with bone or visceral involvement or disease that is refractory to sulfonamides, consideration should be given to intravenous Imipenem with/without amikacin [48]. The optimal duration of mycetoma treatment has not been determined. In immunocompetent patients with cutaneous disease oral therapy for 3 to 6 months is recommended [48]. Immunocompromised patients will need longer treatment for up to 1 year.

In case of severe nocardiosis, despite lack of data to support superiority of combination antibiotic therapy over single drug regimens, most experts recommend initial treatment with two intravenous drugs prior to the availability of susceptibility results [3, 45]. In case of life-threatening infections, three-drug regimen is mostly suggested [45].

In case of severe infection that does not involve the CNS, treatment with TMP-SMX (15 mg/kg IV of the TMP component per day in 3 to 4 divided doses) plus amikacin (7.5 mg/kg IV every 12 hours) is generally recommended [3]. Alternatively imipenem (500 mg IV every 6 hours) in combination with amikacin could be used [3].

In case of patients with isolated CNS disease, TMP-SMX (15 mg/kg IV of the TMP component per day in 3 to 4 divided doses) plus imipenem (500 mg IV every 6 hours) is usually recommended. However, in patients with CNS disease with multiorgan involvement, amikacin should be added to the above-mentioned regimen [3]. Initial treatment or induction therapy should be continued for at least three to six weeks until clinical improvement is documented. Patients who show improvement on three-intravenous-antibiotic regimen and also have* Nocardia* isolates susceptible to treatment could be switched to two intravenous antibiotics to complete the induction phase of therapy. Patients who show signs of clinical improvement could be switched to oral regimen. Recommended antibiotic that can be part of oral regimen include TMP-SMX and/or minocycline and or amoxicillin-clavulanate [3].

Surgical intervention may be necessary in case of cerebral and large soft tissue abscess that do not respond to antibiotics or in case of pulmonary nocardiosis complicated with pericarditis, empyema, and mediastinal fluid collections [24, 51, 52].

In immunosuppressed conditions, such as patients with HIV or on chronic steroids etc., some authorities recommend prolonged oral maintenancetherapy to prevent relapse [49]. TMP-SMX is most commonly used but the protection is not complete [53].

## Figures and Tables

**Table 1:** Jannat-Khah et al. [2] report 4 strains of *Nocardia* that were genotypically similar and named them *Nocardia mikamii* sp. in honor of Dr. Yuzuru Mikami.

*Nocardia* strains collected between 2001 and 2007	Isolate site	Phenotypic differentiating property	Genetic analysis
(1) W7467	Respiratory isolate		100% similar to other 3 strains
(2) W7811	Respiratory isolate	Did not assimilate trehalose	100% similar to other 3 strains
(3) W8061	Respiratory isolate	No aryl sulfatase production at 14 days	100% similar to other 3 strains
(4) W9013	Respiratory isolate	Did not assimilate D galactose	100% similar to other three strains
